# Editorial: New infectious agents in arthropod vectors

**DOI:** 10.3389/fmicb.2022.1105082

**Published:** 2022-12-09

**Authors:** Qiaocheng Chang, Ze Chen, Michael E. von Fricken, Quan Liu

**Affiliations:** ^1^School of Public Health, Shantou University, Shantou, China; ^2^Hebei Key Laboratory of Animal Physiology, Biochemistry and Molecular Biology, College of Life Sciences, Hebei Normal University, Shijiazhuang, China; ^3^Department of Global and Community Health, George Mason University, Fairfax, VA, United States; ^4^Department of Infectious Diseases, Center of Infectious Diseases and Pathogen Biology, The First Hospital, Jilin University, Changchun, China; ^5^School of Life Science and Engineering, Foshan University, Foshan, China

**Keywords:** new infectious agents, virome, metagenome, vertebrate hosts, arthropod vectors

Vector-borne infectious diseases are an important global public health concern, with new pathogens emerging over the past few decades due to the socioeconomic, environmental, and climate change factors (Chala and Hamde, 2021). New detection methods, including virome, meta-transcriptome, metagenome, as well as other specific sequencing and molecular technologies, are widely being deployed for detecting emerging and re-emerging vector-borne pathogens (Angelakis and Raoult, 2014; Epstein and Anthony, 2017). Using these techniques, a large number of new pathogens, including viruses, bacteria, fungi, protozoan and helminth parasites, from both animal hosts and medically relevant vectors, have been discovered (Ma et al., 2021; Zhang et al., 2022). High resolution sequence data generated from these efforts are important for both clinical and public health countermeasures, including prevention, treatment, surveillance, and control. This information combined with biological evidence of transmission can aid in the determination and classification of new infectious agents.

*Colpodella* species are a group of free-living small predatory flagellates related to apicomplexans (Yuan et al., 2012). Under this special topic, Xu et al. reported *Colpodella* spp. infection in horses using PCR assay. *Ixodes persulcatus* may serve as the possible vector for *Colpodella* spp., which is associated with neurological disease in immunocompromised individuals (Jiang et al., 2018). Further investigation should be conducted to determine the roles of horses in the transmission of this new discovered pathogen and to identify the clinical symptoms of horses infected with *Colpodella* spp.. He et al. conducted a thorough and broad-range investigation of *Borrelia burgdorferi* sensu lato in domestic mammals, small wild mammals and ticks collected from Yunnan Province, China. Six genospecies, including *B. burgdorferi* sensu stricto, *B. afzelii, B. garinii, B. japonica, B. sinica*, and *B. valaisiana* were detected positive, indicating a wide distribution of multiple *Borrelia* genospecies in Yunnan, China. Guo et al. investigated the virome of *Rhipicephalus* ticks in Guangdong Province, southern China, and a total of ten viruses, including three phenuivruses, two chuviruses, one each rhabdovirus, flavivirus, orthomyxovirus, and reovirus, and an unclassified virus (Guangdong tick Manly virus), were identified using meta-transcriptome analysis. Importantly, most of these viruses were genetically associated with the viruses reported in Turkey, Trinidad and Tobago, Australia, Thailand, northern Europe, and Brazil, suggesting that it is necessary to continuously survey tick-borne viruses in southern China.

Lin et al. reported high prevalence of *Rickettsia* spp. in *Dermacentor everestianus* and *Haemaphysalis qinghaiensis* ticks collected from yaks in Shiqu county, eastern Tibetan Plateau, China. In this region, the primary mammalian hosts of ticks included domesticated yaks and wild mammals, such as rodents and plateau pika. Lu et al. revealed a high diversity of *Anaplasma, Ehrlichia*, and *Rickettsia* in ticks collected from Yunnan Province, and a novel species of *Rickettsia* was identified. Moreover, most of these bacteria can cause diseases in humans, such as *E. canis, E. chaffeensis*, and *A. ovis*, with high infection rates in these detected ticks. Therefore, there is potential risk of zoonoses transmitted from ticks to humans, resulting from the frequent contact between humans and domestic animals (goats and cattle) that act as tick hosts in these regions. Zawada et al. described high levels of *B. burgdorferi* genetic diversity in white-footed mice, with tongue and ear samples containing 90% of detected subtypes. The work emphasized the need to incorporate metagenomic sequencing into ongoing Lyme disease surveillance efforts.

Alongshan virus (ALSV) is a newly discovered segmented tick-borne flavivirus that can cause human febrile illness (Wang et al., 2019). The viral genome contains four segments and encodes the structural proteins of VP1-VP4 and non-structural proteins of NSP1 and NSP2. Zhao et al. investigated the subcellular distribution and possible functions of viral proteins in host cells, and found that the ALSV proteins had distinct subcellular distribution in different tissue-deriving cells, and caused endoplasmic reticulum morphological changes, and NSP1 could induce mitophagy to reduce mitochondria quantity. These findings help to understand the pathogenic mechanisms of emerging segmented flaviviruses. *Babesia microti*, a tick-transmitted hematoprotozoan, can infect small rodents and humans (Gray and Ogden, 2021). Shu et al. found that *B. microti* can effectively suppress melanoma cell growth and extend the survival time of tumor-bearing mice through increasing the number of CD4+ T cells and macrophages and inducing the conversion of macrophages from type M2 to M1. However, the macrophage activation mechanisms by *B. microti* remains to be explained.

The Topic was intended to focus on new infectious agents in mosquitoes, ticks, fleas, flies, midges, and other blood-sucking arthropods. However, a majority of submitted and all accepted manuscripts focused on tick-borne pathogens in China. These results suggest a high diversity of tick-borne pathogens in China, especially viruses and bacteria, whose epidemiology, biology and public health significance should be further investigated. Moreover, further Research Topics specific to other medically relevant arthropods vectors is recommended.

## Author contributions

QL and QC drafted the manuscript. All authors corrected, edited, and approved the manuscript. All authors listed have made a substantial, direct, and intellectual contribution to the work and approved it for publication.
